# Association of post-stroke-initiated antidepressants with long-term outcomes in young adults with ischaemic stroke

**DOI:** 10.1080/07853890.2022.2089729

**Published:** 2022-07-04

**Authors:** Jenna Broman, Karoliina Aarnio, Anna But, Ivan Marinkovic, Jorge Rodríguez-Pardo, Markku Kaste, Turgut Tatlisumak, Jukka Putaala

**Affiliations:** a Department of Neurology, Helsinki University Hospital and University of Helsinki, Helsinki, Finland; b Department of Public Health, University of Helsinki, Helsinki, Finland; c Department of Neurology, La Paz University Hospital, Madrid, Spain; d Department of Clinical Neuroscience, Institute of Neuroscience and Physiology, Sahlgrenska Academy at University of Gothenburg, Gothenburg, Sweden; e Department of Neurology, Sahlgrenska University Hospital, Gothenburg, Sweden

**Keywords:** Antidepressants, brain infarction, stroke, young adult, prognosis

## Abstract

**Objective:**

We examined the association between initiation of antidepressants within the first year after ischaemic stroke (IS) in young adults and long-term fatal and non-fatal cardiovascular events, as well as all-cause mortality.

**Patients and methods:**

The Helsinki Young Stroke Registry (HYSR) includes patients aged 15–49 years with their first-ever IS occurring 1994–2007. From nationwide registers, we obtained data on prescriptions (1993–2011) and outcomes of interest (1994–2011). Time of initiating post-stroke antidepressants (PSADs) was defined as time of the first filled prescription for antidepressants within the first year from IS. To account for non-random assignment of PSADs, we performed propensity score matching and studied the relationship between PSAD initiation and outcomes using Cox regression models with time-varying coefficients.

**Results:**

Of all patients (*n* = 888), 206 (23.2%) initiated PSADs within the first year, of which 203 (98.5%) could be matched to 406 non-initiators. In this matched sample of 609 patients, the median follow-up time was 8.1 (interquartile range [IQR] 5.0–12.6) years and 169 (28.9%) patients had any cardiovascular events, 95 (15.8%) had recurrent ischaemic or haemorrhagic strokes and 106 (17.4%) died. Adjusted for sociodemographics and cardiovascular comorbidities, PSAD initiation was associated with recurrent ischaemic or haemorrhagic stroke 5–10 years after IS (hazard ratio [HR] 3.07, 95% confidence interval [CI] 1.32–7.12). No association emerged between PSAD initiation and other outcomes.

**Conclusions:**

In young adults, PSAD initiation within the first year after IS was associated with a heightened hazard of recurrent ischaemic or haemorrhagic stroke in the long term. Future studies are needed to verify the results and to further study the nature of this finding.

**KEY MESSAGES**
- Initiation of post-stroke antidepressants (PSADs) within the first year after ischaemic stroke (IS) was associated with a heightened hazard of recurrent ischaemic or haemorrhagic stroke in the long term.
- Patients starting antidepressants after IS should be followed up more closely in case of recurrent events.
- Future studies are needed to verify the results and to further study the nature of this finding.

## Introduction

Post-stroke depression affects nearly one-third of stroke patients, influencing the individuals’ quality of life, as well as worsening their outcomes [1–6]. This seems to apply to young patients as well [7], among whom post-stroke depression could have a notable effect on their vocational performance, family relations and health in general.

In most patients, symptoms of depression develop shortly after stroke [2,6]. In addition to lower quality of life as well as reduced activities of daily living, post-stroke depression is known to be associated with impaired functional recovery, increased disability and mortality [2,6,8–13]. Post-stroke depression is known to also be associated with long-term mortality among young IS patients [14]. Existing studies have mostly focussed on older patients and suggest that even the use of antidepressants after stroke might be associated with higher mortality and other unfavourable outcomes, including higher risk of stroke recurrence, although findings are controversial [8,15,16].

There are limited data on the consequences of newly initiated post-stroke antidepressants (PSADs), especially in the young. In our previous study [17], we observed an association between several specific clinical characteristics and initiation of PSADs. In this study, we aimed to assess the association of initiation of PSADs with all-cause mortality, a composite of any vascular event and recurrent ischaemic or haemorrhagic stroke among young adults with their first-ever IS.

## Patients and methods

### Study population

The cohort of this register-based follow-up study originates from the Helsinki Young Stroke Registry (HYSR), including 1008 consecutive patients aged 15–49 years with their first-ever ischaemic stroke (IS) who were treated at the Department of Neurology, Helsinki University Hospital between January 1994 and May 2007, as identified from a prospective computerized hospital discharge database. The original World Health Organization stroke definition was utilized, however, patients with a short duration of symptoms when imaging-positive findings of IS were present were also included [18]. Transient ischaemic attacks were excluded. By using the personal identification number assigned to every resident in Finland, the data from HYSR were further combined with data obtained from several national registers. From this study, patients with a false primary diagnosis (*n* = 4), those not able to be linked to databases (*n* = 14), those dying within 3 weeks from IS (i.e. in-hospital deaths) (*n* = 24), and those who had filled a prescription for antidepressants within one year prior to index stroke and thus could not be considered as newly initiating PSADs during the follow-up (*n* = 78) were excluded. After applying these exclusion criteria, the study population included 888 patients with a first-ever IS [17].

### Ethics approval

The Ethics Committee of the Hospital District of Helsinki and Uusimaa approved the study protocol (73/13/03/00/11). Since this study is a continuation of the same research project including many prior studies performed on the HYSR, the Ethics approval number remains the same as in our previous study [17].

### Consent to participate

Informed consent from patients in our cohort was not needed because the study is based on registry data without direct patient contact.

### Baseline data

Baseline laboratory as well as other diagnostic tests has already been fully presented previously [19]. All patients underwent a chest x-ray, ECG and brain imaging with computed tomography (CT) or magnetic resonance imaging (MRI). Data on patients’ sociodemographics, including age, sex and socioeconomic status [20]; data on IS risk factors, including the status of cigarette smoking at the time of index event, heavy alcohol use described as consumption over 200 g a week, cardiovascular disease, atrial fibrillation, hypertension, dyslipidaemia, diabetes mellitus types 1 and 2; data on stroke-related variables assessed at hospital admission, including NIH Stroke Scale (NIHSS), Trial of Org 10172 in Acute Stroke Treatment (TOAST) classification and silent infarcts; and data on limb paresis at hospital discharge were collected.

We classified the patient’s socioeconomic status based on occupation as: upper white collar worker, lower white collar worker, blue collar worker, other (entrepreneur, student, pensioner and unemployed) or unknown and missing [20]. Stroke severity was classified as mild (NIHSS 0–6), moderate (NIHSS 7–14) and severe (NIHSS ≥15), based on NIHSS score at admission. Categories for limb paresis at hospital discharge were: no paresis, mild (1 point from NIHSS sections 5–6) and moderate to severe (2–4 points from NIHSS sections 5–6).

### Follow-up data

Data on the outcomes of interest occurring 1994–2011 as well as dates and diagnoses for psychiatric hospitalizations during the follow-up [17] were obtained from the Care Register for Health Care [21], and all possible cases of composite vascular events identified from this register were also verified from patient records (*n* = 165, 97.6%) [21]. Dates and causes of death came from Statistics Finland. The follow-up started at the index stroke and ended at the occurrence of the event of interest, death, or on 31 December 2011, whichever came first.

Endpoints of interest were: all-cause mortality, a composite of any nonfatal or fatal vascular event and nonfatal and fatal recurrent ischaemic or haemorrhagic stroke. The composite endpoint included recurrent strokes (ischaemic and haemorrhagic) and other vascular events; cardiac events (acute coronary syndromes, cardiac death and other cardiac events), peripheral arterial events and venous events including pulmonary embolism and deep vein thrombosis. The events’ International Classification of Diseases codes and respective clinical definitions have already been previously published [21]. Transient ischaemic attacks were excluded.

Data on prescriptions filled between 1993 and 2011 and purchase dates were obtained from the Drug Prescription Register kept by the Social Insurance Institution of Finland, including Anatomical Therapeutic Chemical (ATC) codes (WHO Collaborating Centre for Drug Statistics Methodology, 2018). Information on all prescriptions assigned by any physician in Finland is included in this register. Prescribed medications in Finland are entitled to reimbursement of 40–100% of the medicine’s price and the Social Insurance Institution of Finland can only reimburse a 3 months’ equivalent amount of medication at any one time. We identified patients who had filled prescriptions of antidepressants prior to and post index IS based on ATC code N06A [17]. In this study, PSAD initiators were defined as patients with at least one filled prescription of antidepressant medication within the first year after index IS, therefore emulating an intention-to-treat approach. Person-time contributed by patients who initiated PSADs was classified as time on PSADs regardless of the exact time the first prescription of antidepressants was filled. Those patients who initiated PSADs after the event of interest (eight patients with vascular events and two with recurrent stroke) were excluded from the respective analysis along with their matches (altogether 24 and six patients, respectively).

### Statistical analyses

The baseline characteristics of the patients are presented with descriptive statistics, stratified by PSAD initiation. We reported medians and interquartile ranges (IQRs) for continuous variables, and numbers of observations and percentages for categorical variables. Imbalance in the baseline characteristics between patients with and without PSADs was assessed by means of average absolute standardized difference. Missing data were reported as number of observations and percentages.

Because PSAD initiators and non-initiators differed with respect to baseline characteristics [17], and PSADs were not randomly assigned in the study population, we used propensity score matching, which allows for reducing the risk of bias owing to confounding due to non-random allocation and estimating the average treatment effect for the treated (i.e. the average effect of PSAD initiation on those who ultimately purchased PSADs) [22]. The nearest neighbour method was used to match PSAD initiators to non-initiators (ratio 1:2) based on the logit of the propensity score from the logistic regression model including the covariates identified to be associated with PSAD initiation [17]. We examined balance in the baseline characteristics in the matched sample by means of average absolute standardized difference. In addition, we graphically inspected the distribution of propensity scores among PSAD initiators and non-initiators before and after matching.

As recommended by Austin et al. [23], to account for competing risks potentially involved, we assessed subdistribution estimates of cumulative incidence rather than the complement of the Kaplan–Meier survival estimate. Separately for any vascular event and for restroke, we examined the cumulative incidence of fatal and non-fatal events of interest, when taking into account death from other causes. We presented the resulting estimates graphically and reported the 5-, 10- and 15-year cumulative incidence with 95% confidence intervals (CIs).

For each outcome of interest, we fitted univariable Cox regression models on the matched sample. Since the assumption of proportional hazards was not satisfied for the effect of the PSAD initiation, we split the follow-up time into three different time strata (0–5 years, 5–10 and >10) to allow for time-varying hazard ratios (HRs). To account for clustering within matched sets (lack of independence), we used a robust variance estimator (Huber sandwich estimator). For each outcome, we reported HRs and their corresponding 95% CIs in each time stratum. Although matching accounts for confounding, matching can also be combined with regression adjustment to reduce bias due to residual differences. We constructed four different multivariable Cox regression models to adjust for factors previously identified as potential confounders [8,15–17,24] and/or factors satisfying the definition of a confounder as based on association with both the exposure and the outcome, and given the assumed relationships between the factors [25]. We considered age, sex and socioeconomic status as essential to control for in the models. Thus, the first model was adjusted for age, sex and socioeconomic status. In addition to these demographic factors, the second model was further adjusted with stroke characteristics (NIHSS, TOAST classification, silent infarcts and limb paresis at discharge), the third model was further adjusted for lifestyle factors (current smoking and heavy alcohol use) and the fourth, fully adjusted model was further adjusted for other cardiovascular comorbidities (atrial fibrillation, hypertension, dyslipidaemia, cardiovascular disease and diabetes mellitus types 1 and type 2). As a sensitivity analysis, the time stratum of 0–5 years was changed to 1–5 years to check whether the results remain the same when excluding the period that is subject to misclassification of person time.

Statistical analyses were performed with the R software (R Core Team; R Foundation for Statistical Computing, Vienna, Austria) [26], including packages MatchIt and cmprsk.

## Results

From the dataset of 888 patients, we excluded 14 (2.1%) PSAD non-initiators and 3 (1.5%) initiators with missing data on socioeconomic status, as well as 8 (1.2%) PSAD non-initiators with missing data on limb paresis at discharge, resulting in a final dataset of 863 patients. From a total of 206 PSAD initiators, 203 (98.5%) patients could be matched to 406 PSAD non-initiators. Table 1 shows the baseline characteristics of the PSAD non-initiators and initiators, and the standardized errors in the original (*n* = 660) and matched sample (*n* = 406). The standardized mean difference values became closer to zero after propensity score matching, indicating the matched sample to be more balanced (Table 1). Supplemental Figure 1 presents the distribution of propensity scores among PSAD initiators and non-initiators before and after matching. Overlapping of the distribution of propensity scores improved substantially through propensity score matching, except in patients with propensity scores > 0.7, of which 17 of 22 were PSAD initiators. Altogether, 25 (12.8%) of the PSAD initiators had at least one antidepressant purchase within a year prior to the vascular event, 17 (8.5%) prior restroke and 19 (9.4%) prior death.

**Table 1.** Characteristics of the PSAD non-initiators and initiators and standardized mean differences of variables in the original non-matched and matched sample.

Characteristic	Not initiated PSAD				Initiated PSAD *n* = 203
	Original sample^a^*n* = 660 (76.5%)		Matched sample *n* = 406 (66.7%)		
	*n* (%) or median (IQR)	SMD	*n* (%) or median (IQR)	SMD	*n* (%) or median (IQR)
Sociodemographic variables					
Age at IS, years	43.0 (36.0–47.0)	0.192	45.0 (38.0–47.0)	0.045	44.0 (38.5–47.0)
Male sex	428 (64.8)	0.098	248 (61.1)	0.020	122 (60.1)
Socioeconomic status		0.208		0.017	
Upper white-collar worker	74 (11.2)		58 (14.3)		25 (12.3)
Lower white-collar worker	153 (23.2)		119 (29.3)		61 (30.0)
Blue collar worker	299 (45.3)		131 (32.3)		69 (34.0)
Other/unknown	134 (20.3)		98 (24.1)		48 (23.6)
Prior antidepressant use (earlier than one year)^b^	41 (6.2)	0.282	39 (9.6)	0.158	30 (14.8)
Risk factors for IS					
Comorbidities					
Atrial fibrillation	23 (3.5)	0.002	15 (3.7)	0.013	7 (3.4)
Cardiovascular disease	61 (9.2)	0.069	40 (9.9)	0.048	23 (11.3)
Diabetes mellitus type 1	30 (4.5)	0.018	18 (4.4)	0.023	10 (4.9)
Diabetes mellitus type 2	42 (6.4)	0.019	31 (7.6)	0.069	12 (5.9)
Dyslipidaemia	388 (58.8)	0.047	244 (60.1)	0.020	124 (61.1)
Hypertension	254 (38.5)	0.109	157 (38.7)	0.105	89 (43.8)
Lifestyle factors					
Current cigarette smoking	277 (42.0)	0.156	198 (48.8)	0.020	101 (49.8)
Heavy alcohol drinking	82 (12.4)	0.041	60 (14.8)	0.028	28 (13.8)
Stroke-related variables measured at hospital admission					
NIHSS at admission		0.617		0.402	
0–6, mild	548 (83.0)		299 (73.6)		109 (53.7)
7–14, moderate	76 (11.5)		73 (18.0)		61 (30.0)
≥15, severe	36 (5.5)		34 (8.4)		33 (16.3)
Silent infarcts	73 (11.1)	0.203	64 (15.8)	0.065	37 (18.2)
TOAST		0.097		0.057	
Large-artery atherosclerosis	40 (6.1)		30 (7.4)		24 (11.8)
Cardioembolism	125 (18.9)		74 (18.2)		34 (16.7)
Small-vessel disease	100 (15.2)		68 (16.7)		22 (10.8)
Other	164 (24.8)		97 (23.9)		61 (30.0)
Undetermined causes	231 (35.0)		137 (33.7)		62 (30.5)
Disability at discharge					
Limb paresis at discharge		0.789		0.535	
No	518 (78.5)		271 (66.7)		92 (45.3)
Mild	84 (12.7)		77 (19.0)		36 (17.7)
Moderate–severe	58 (8.8)		58 (14.3)		75 (36.9)

PSAD: post-stroke antidepressant; IS: ischaemic stroke; NIHSS: NIH Stroke Scale; TOAST: Trial of Org 10172 in Acute Stroke Treatment; SMD: standardized mean difference.

^a^In the original sample, 25 (2.8%) patients were excluded due to missing values in socioeconomic status and limb paresis at discharge.

^b^PSAD purchased earlier than a year prior to IS.

**Figure 1.**
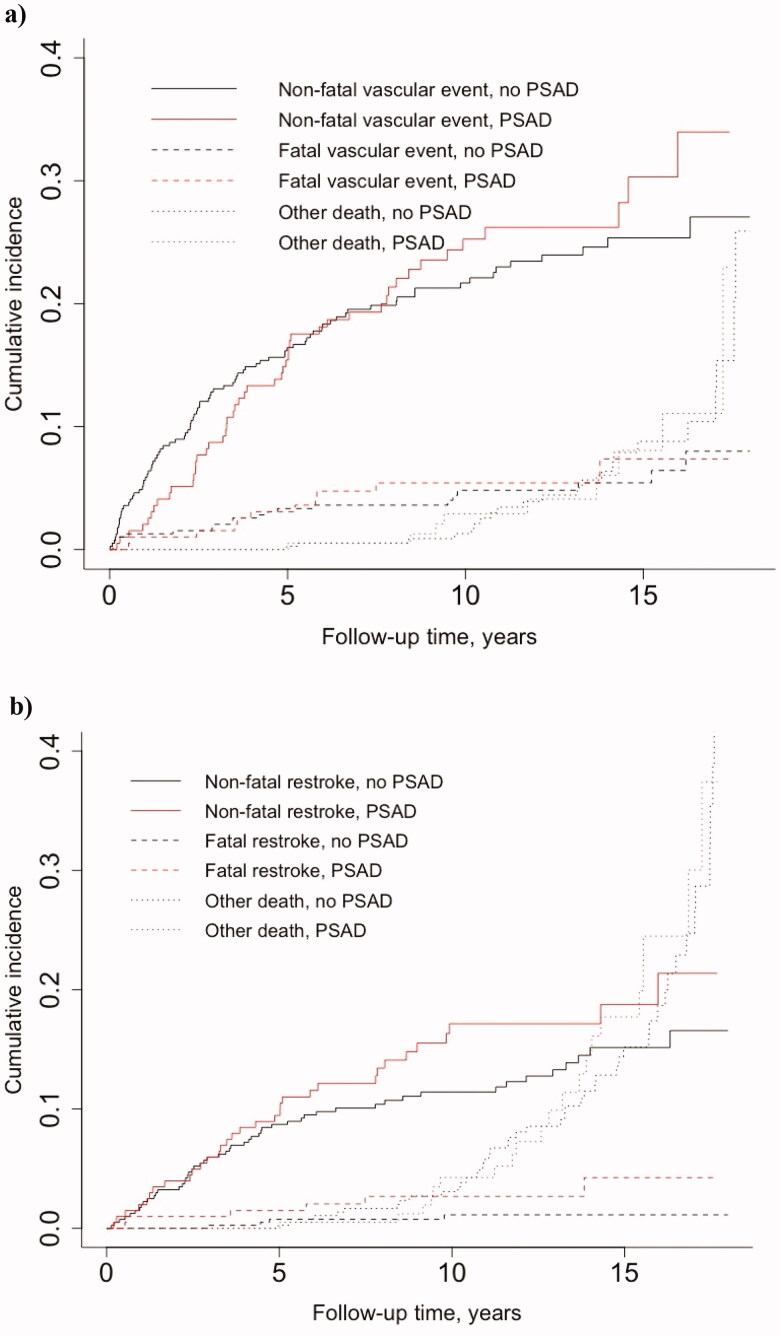
Estimated cumulative incidence curves in competing risks analyses for a) non-fatal vascular event *vs*. vascular and non-vascular mortality occurring before vascular event and b) non-fatal restroke *vs*. restroke and non-restroke mortality occurring before restroke in patients initiating post-stroke antidepressants (PSAD) within one year from ischaemic stroke. Eight PSAD initiators and 16 matched controls were excluded from the vascular event dataset (*n* = 585), and 2 initiators and 4 matched controls were excluded from the recurrent stroke dataset (*n* = 603), since in these cases the PSAD use started after the event of interest.

In the matched sample of 609 patients, during a median follow-up of 8.1 (IQR 5.0–12.6) years, a total of 5172 person-years accumulated for 609 patients when followed until death or end of follow-up, yielding a crude mortality rate of 17.0 per 1000 person-years. Supplemental Table 1 shows the corresponding numbers for composite of any cardiovascular event, recurrent ischaemic or haemorrhagic stroke and mortality. For any (non-fatal or fatal) vascular event, the 10-year cumulative incidence was similar in PSAD initiators (30.7%, 95% CI 20.8–40.5) and non-initiators (26.5%, 95% CI 20.0–33.0). Similarly, we observed no difference in the 10-year cumulative incidence of non-vascular mortality between PSAD initiators (2.9%, 95% CI 0.05–5.8) and non-initiators (1.3%, 95% CI 0.007–2.6). We found that the 10-year cumulative incidence of non-fatal or fatal recurrent stroke in PSAD initiators was 19.8% (95% CI 11.8–27.8), whereas in non-initiators 12.5 (95% CI 8.2–16.9). There was no difference in the 10-year cumulative incidence rate for non-restroke death in PSAD initiators (4.3%, 95% CI 0.9–7.6) and non-initiators (3.1%, 95% CI 1.2–5.0) (Supplemental Table 2 and Figure 1).

**Table 2.** Association between initiation of post-stroke antidepressants and mortality, any vascular event and recurrent stroke.

	Univariate	Model 1	Model 2	Model 3	Model 4
	PSADs within one year	Adjusted for age, sex and socioeconomic status	Adjusted forModel 1 + NIHSS, TOAST, silent infarct and limb paresis	Adjusted for Model 2 + smoking and heavy alcohol drinking	Adjusted for Model 3 + cardiovascular comorbidities^a^
	HR (95% CI)	aHR (95% CI)	aHR (95% CI)	aHR (95% CI)	aHR (95% CI)
Mortality (*n* = 609)					
0–5 years	0.86 (0.46–1.62)	0.83 (0.43–1.59)	0.63 (0.31–1.26)	0.66 (0.33–1.31)	0.63 (0.31–1.30)
5–10 years	0.85 (0.43–1.70)	0.79 (0.40–1.59)	0.55 (0.25–1.20)	0.58 (0.26–1.28)	0.56 (0.25–1.23)
>10 years	1.59 (0.65–3.86)	1.30 (0.55–3.08)	0.83 (0.35–1.99)	0.88 (0.36–2.10)	0.84 (0.35–2.04)
Any vascular event (*n* = 585)^b^					
0–5 years	0.90 (0.63–1.30)	0.90 (0.62–1.29)	0.92 (0.63–1.35)	0.91 (0.62–1.34)	0.88 (0.60–1.30)
5–10 years	1.91 (1.05–3.50)	1.85 (1.00–3.43)	1.87 (1.00–3.50)	1.85 (0.98–3.50)	1.76 (0.93–3.32)
>10 years	1.24 (0.43–3.61)	1.05 (0.38–2.91)	1.06 (0.36–3.08)	1.00 (0.34–2.91)	1.04 (0.35–3.06)
Recurrent ischaemic or haemorrhagic stroke (*n* = 603)^b^					
0–5 years	1.17 (0.71–1.94)	1.19 (0.72–1.96)	1.22 (0.72–2.09)	1.19 (0.69–2.05)	1.27 (0.74–2.20)
5–10 years	2.99 (1.34–6.65)	2.93 (1.29–6.65)	2.95 (1.29–6.74)	2.91(1.26–6.68)	3.07 (1.32–7.12)
>10 years	0.99 (0.25–3.93)	0.92 (0.24–3.45)	0.92 (0.23–3.65)	0.90 (0.22–3.61)	1.00 (0.25–4.02)

PSAD: post-stroke antidepressant; HR: hazard ratio; aHR: adjusted hazard ratio; CI: confidence interval; NIHSS: NIH Stroke Scale; TOAST: Trial of Org 10172 in Acute Stroke Treatment.

^a^Including variables atrial fibrillation, hypertension, dyslipidaemia, cardiovascular disease and diabetes mellitus type 1 and type 2.

**^b^**Eight PSAD initiators and 16 matched controls were excluded from the vascular event dataset (*n* = 585), and 2 initiators and 4 matched controls were excluded from the recurrent stroke dataset (*n* = 603), since in these cases the PSAD was initiated after the event of interest.

PSAD initiation was associated with an increased hazard for recurrent ischaemic or haemorrhagic stroke 5–10 years after IS in both univariate and all multivariable Cox regression models (Table 2). Furthermore, non-fatal recurrent strokes mainly contributed to the differences seen for this time period (Supplemental Table 2 and Figure 1). Initiation of PSADs was associated with increased hazard for composite of any cardiovascular event only univariately, and there was no association between PSAD initiation and mortality (Table 2). In sensitivity analysis, there was no statistically significant change in the results when the time stratum of 0–5 years was changed to 1–5 years (data not shown).

## Discussion

The effects of newly initiated PSADs have been poorly characterized, especially in young IS patients. In our registry-based matched sample of 609 young adults with their first-ever IS, we found an association between PSAD initiation within the first year after IS and a long-term increased rate of recurrent ischaemic or haemorrhagic stroke 5–10 years after IS. Interestingly, we observed no association between PSAD initiation and recurrent stroke within the first 5 years after index IS. We found no differences in all-cause mortality and the hazard rate of any vascular event between PSAD initiators compared with non-initiators.

A large Danish cohort study reported that stroke patients (ischaemic and haemorrhagic stroke or transient ischaemic attack) aged 15 years and older had higher incidence of depression within 3 months from hospitalization when compared with the reference population [6]. Furthermore, post-stroke depression is known to be associated with higher all-cause mortality and this is also the case among young IS patients [4,6,8,11,14]. In the Danish study, depression was associated with higher all-cause mortality rates in both stroke patients and controls, but the relative mortality was lower in stroke patients [6]. A study on mostly older patients from the South London Stroke Register in 1997–2010 also found that depression at 3 months after first-ever ischaemic or haemorrhagic stroke was associated with increased mortality rates up to 5 years after stroke [4]. Even if the patient recovered from depression, mortality rates remained higher at 5 years’ follow-up compared to those not having depression. Furthermore, depression at 5 years after stroke was not associated with higher mortality up to 10 years follow-up. The authors hypothesized that post-stroke depression shortly after the index event may be a more important predictor of long-term stroke prognosis than depression later on is [4]. This study did not observe any associations between depression and recurrent stroke as we did between PSAD initiation and restroke in our study. Furthermore, cardiovascular comorbidities were not taken into account when assessing the relationship between depression and outcome events, and associations between depression after stroke and other recurrent cardiovascular events were not studied [4].

A systematic review and a meta-analysis of prospective cohort studies examined the association between depression in general and future risk of developing stroke and indicated that depression is associated with increased risk of total stroke, as well as fatal and IS [24]. Furthermore, a positive association was found between antidepressant medication use and risk of stroke, although the result might also be an indication of more severe depression [24]. Other studies have also suggested that the use of antidepressants in general may be associated with an increased risk of cardiovascular and cerebrovascular disease [27], and that antidepressant use after IS could be associated with increased mortality and lead to other unfavourable outcomes, including a higher risk of recurrent strokes [8,15,16]. While our study found no association between PSAD initiation and short- and long-term all-cause mortality, in another study, selective serotonin reuptake inhibitor (SSRI) use started after stroke was independently associated with higher mortality within 5 years of stroke [8]. However, this study evaluated the use of SSRIs at three months after stroke in patients with depression, whereas data on clinical depression diagnosis were not available in our study. Another Danish population-based follow-up study on patients with a mean age of 72 years and including data from 1995 onward with a median follow-up time of over 3 years also found that SSRI medication may be associated with increased mortality in patients with IS, but concluded that these results may reflect a combination of uncontrolled confounding by indication owing to the underlying depression and an increased bleeding risk [15]. Nonetheless, two recent randomized, placebo-controlled, double blind, parallel group trials including mainly older ischaemic and haemorrhagic stroke patients found no benefit on functional outcome with SSRI treatment following stroke nor an increase in mortality, new strokes or haemorrhagic events after six months from the index event [28,29].

On the other hand, the Danish study also found that the risk of acute myocardial infarction and recurrent IS decreased with antidepressant treatment after stroke [15]. However, data on antidepressant use and its association with recurrent strokes are limited [15,16]. A cohort study from Taiwan found that the use of antidepressants in patients aged ≥ 20 years and with ischaemic or haemorrhagic stroke was associated with a 40% greater risk of stroke recurrence, especially in IS patients [16]. We also found that antidepressants started after IS was associated with an increased hazard for recurrent stroke within the time period of 5–10 years after IS. We hypothesize that this observation may stem from possible diminished adherence to IS secondary prevention measures after the first years from IS possibly owing to more severe depression, or these patients might represent a population with different susceptibility to recurrent stroke owing to possibly different aetiologies with a higher risk of recurrence, such as large-artery atherosclerosis and cardioembolism [30]. Another possible explanation might be residual confounding due to changes in both the population at risk and risk factors for restroke during the follow-up [30]. It is possible that these changes might have occurred in a different way among PSAD initiators compared to non-initiators. In addition, although not statistically significant, the lower vascular mortality rates in PSAD initiators within the first 5 years could also reflect that these patients have better compliance to treatment in general. Adherence to treatment in turn can be associated with disability severity within the first year from IS and vice versa, untreated depression can affect patient compliance and disability. Finally, initiation of antidepressants after IS could thus be an indicator of generally more vulnerable patients for recurrent strokes, and anyhow these patients should be followed up more closely in case of recurrent events.

The several strengths of this study include a large study population of young IS patients with detailed data on baseline stroke characteristics and other clinically significant factors. In addition, the follow-up data from the Finnish registers, which are known to be of good to high quality [31,32], reduced information bias and allowed us to evaluate both the short- and long-term outcomes of interest. The outcome data were also mostly verified from patient records, hence the chance of outcome misclassification was even smaller [21]. Compared to clinical trials, our observational study provides the benefit of being in a real-life setting. However, our purpose was also to emulate randomized controlled trials and evaluate and reduce potential biases arising in observational studies. Thus, we used advanced statistical methods, such as propensity score matching and Cox models with time-varying coefficients. We studied the cumulative incidence by accounting for death from other causes as a competing risk and found no difference between PSAD initiators and non-initiators in cumulative mortality. In order to emulate an intention-to-treat approach, to avoid potential effects of recent antidepressant use, and to assess the association of newly initiated (i.e. most likely stroke-related) PSAD with long-term outcomes, we excluded patients with antidepressant purchase within one year prior to index event and accounted for only PSADs started within the first year after IS. We excluded 24 patients who died within the first 21 d after IS to include only those who survived the primary hospitalization (i.e. patients who had at least the theoretical possibility to initiate PSADs). All of these excluded patients died within the in-hospital period, most of them (*n* = 20) during the first week of the index event.

There are also some unavoidable limitations present in this study, and some sources of bias are difficult to control completely due to the retrospective observational nature of the study. The misclassification of person-time may arise due to the fixed binary representation of PSAD initiation we used, which did not take into account the exact time of the first PSAD purchase. However, this misclassification is likely to have only a minor effect on the results, especially on the association of PSAD initiation with the long-term outcomes. In fact, only 66 (4%) person-years of the 5172 person-years accumulated during the first 5 years were misclassified. Furthermore, we observed no difference in the results when excluding the period that is subject to misclassification of person-time. Immortal time bias might be present since the follow-up time started at the index event. However, only 12 (3%) of those classified as PSAD non-initiators died within the first year after index IS. Furthermore, the median time for the first PSAD purchase was 101 d (IQR 50–161), and of these 12 patients, 6 died within 64 d from the index event and the rest after 100 d.

Propensity score methods are proposed as a potential solution for taking into account the non-random allocation of treatment. Regardless of propensity score matching used in this study, some differences remained between PSAD initiators and non-initiators at baseline. Particularly, according to prior antidepressant use, NIHSS at admission and limb paresis at discharge, PSAD initiators seemed to be in poorer health at baseline. Moreover, matching by propensity scores cannot correct residual confounding due to unmeasured confounders, changes in confounders with time and healthy user bias. Compared with randomized controlled trials, use of the propensity score method may also overestimate the effect, although the differences are suggested to be rarely statistically significant [33].

We used a quite liberal definition of starting PSADs, as we required only one purchase. However, we found it almost as good as using a stricter definition requiring at least 2 or even more purchases, since only 22 (11%) of the patients whom we classified as PSAD initiators had only 1 purchase. In this register-based study, it was not possible to ensure whether the purchase was followed by actual use of the medication or to assess the compliance with treatment. Therefore, we did not aim to assess the effect of possible discontinuation either. Possible reasons for discontinuing a newly started antidepressant treatment might include, for example, experienced side effects or other unsuitability of the medication. Additionally, discharge to institutional rehabilitation [17] or new hospitalization soon after the initial one can potentially affect patients’ opportunities to purchase PSADs. However, more than half of those initiating PSADs filled their second prescription within 3 months and over three-quarters within 6 months [17], thus indicating that those patients were likely to also become long-term users. Furthermore, 13% of the PSAD initiators purchased antidepressants within a year prior to the vascular event, and 9% prior to restroke and death. Furthermore, we were unable to distinguish the indication for PSADs: whether it was for depression, enhancement of neuroplasticity aiming at improving stroke recovery, or some other reason. We found that, of the PSAD initiators, 13 (6.4%) had a hospitalization due to a mood disorder, including depression at some point after the index IS. However, the total amount of patients with post-stroke depression could not be reliably verified due to the lack of data on outpatient diagnoses. It is also possible that patients with poor adherence to treatment did not have any filled prescriptions despite their depression diagnosis. Furthermore, indication bias might be present, where the risk of adverse endpoint events is related to the indication of antidepressant initiation, for example post-stroke depression, rather than the antidepressant itself. Also, it is shown by prior studies [34], that stroke-induced inflammation may be involved in the process of fatigue and depression after stroke. Our data includes only limited number of inflammation markers (leukocyte count and C-reactive protein) measured at admission, thus containing several possible sources of bias. Therefore, we were not able to assess reliably their role as a confounder. For these reasons, interpretation of the observed association is a challenge.

Finally, the statistical power is limited due to the large number of variables fitted in the progressively adjusted Cox regression models given the number of events of interest and the wide CIs of the HRs for recurrent stroke. However, using progressive adjusting makes the results of different models more comparable. We performed multiple tests to assess the relationship between PSAD initiation and the outcomes of interest, therefore increasing the risk of drawing false-positive conclusions.

In conclusion, patients starting antidepressants after IS, and thus possibly suffering from post-stroke depression, should be followed up more closely in case of future recurrent events. In young adults, PSAD initiation within the first year after IS was associated with a heightened hazard of recurrent ischaemic or haemorrhagic stroke in the long term. However, to verify this association, and to further assess the nature of our finding, future studies on larger populations of young patients with IS comprising both detailed baseline and follow-up information on risk factors are needed.

## Supplementary Material

Supplemental Material

## Data Availability

We have documented the data, methods, and materials used to conduct the research in this report. The individual patient data are not publicly available due to legal restrictions. The syntax code for study analyses in R software [26] is available from the corresponding author, J.B., upon reasonable request.
