# Supplementary material for: Association of post-stroke-initiated antidepressants with long-term outcomes in young adults with ischaemic stroke
Source: Ann Med. 2022 Jul 4;54(1):1757–66. doi: 10.1080/07853890.2022.2089729 (PMC9258433; doi:10.1080/07853890.2022.2089729)

Supplemental data.

For Peer Review Only

Supplemental Table 1. Number of patients at risk, number of events, person-years and crude incidence rates with 95% confidence intervals of a) all-cause, vascular, and restroke mortality, b) vascular event subgroups, and c) recurrent stroke subgroups by PSAD initiated before the outcome of interest.

a)

	All-cause mortality		Vascular mortality		Restroke mortality	
	No PSAD n=406	PSAD n=203	No PSAD n=406	PSAD n=203	No PSAD n=406	PSAD n=203
Number of events	72	34	20	11	4	6
Person-years	4174.8	2045.0	4174.8	2045.0	4174.8	2045.0
Incidence rate[a] (95% CI)	17.2 (13.5–21.7)	16.6 (11.5–23.2)	4.8 (2.9–7.4)	5.4 (2.7–9.6)	0.96 (0.26–2.5)	2.9 (1.1–6.4)

b)

	Any vascular event		Cardiac event		Peripheral arterial event		Venous event	
	No PSAD n=390[b]	PSAD n=195[b]	No PSAD n=390[b]	PSAD n=195[b]	No PSAD n=390[b]	PSAD n=195[b]	No PSAD n=390[b]	PSAD n=195[b]
Number of events	109	60	59	29	21	9	10	5
Person-years	3584.8	1703.0	3584.8	1703.0	3584.8	1703.0	3584.8	1703.0
Incidence rate[a] (95% CI)	30.4 (25.0–36.7)	35.2 (26.9–45.4)	16.5 (12.5–21.2)	17.0 (11.4–24.5)	5.9 (3.6–9.0)	5.3 (2.4–10.0)	2.8 (1.3–5.1)	2.9 (0.95–6.9)

c)

	Recurrent stroke		Ischemic restroke		Hemorrhagic restroke	
	No PSAD n=402[b]	PSAD n=201[b]	No PSAD n=402[b]	PSAD n=201[b]	No PSAD n=402[b]	PSAD n=201[b]
Number of events	56	39	49	35	6	4
Person-years	4213.3	1949.6	4213.3	1949.6	4213.3	1949.6
Incidence rate[a] (95% CI)	13.3 (10.0–17.3)	20.0 (14.2–27.3)	11.6 (8.6–15.4)	18.0 (12.5–25.0)	1.4 (0.52–3.1)	2.1 (0.56–5.3)

PSAD = post-stoke antidepressant; CI = confidence interval.

[a]Per 1000 person-years.

[b]Eight PSAD initiators and 16 matched controls were excluded from the vascular event dataset (n=585), and 2 initiators and 4 matched controls were excluded from the recurrent stroke dataset (n=603), since in these cases the PSAD use started after the event of interest.

Email: IANN-peerreview@journals.tandf.co.uk URL: http://mc.manuscriptcentral.com/sann

For Peer Review Only

Supplemental Table 2. The estimated cumulative incidence rates (95% confidence interval) of any vascular event and restroke as divided into non-fatal and fatal events and assessed by accounting for death from other causes than that of interest in patients with PSAD initiated within the first year from IS.

**PSAD**	**Cumulative incidence % (95% CI)**		
	**5-year**	**10-year**	**15-year**
**Vascular event[a]**			
	**Non-fatal (n=138)**		
Non-initiators	16.4 (12.7–20.1)	21.7 (17.5–25.9)	25.4 (20.5–30.2)
Initiators	15.4 (10.3–20.5)	25.3 (18.7–31.8)	30.3 (21.8–38.8)
	**Fatal (n=31)**		
Non-initiators	3.3 (1.6–5.1)	4.8 (2.5–7.1)	5.4 (2.9–8.0)
Initiators	3.1 (0.6–5.5)	5.4 (2.1–8.7)	7.4 (2.4–12.4)
	**Non-vascular death (n=31)**		
Non-initiators	NA	1.3 (0.007–2.6)	8.8 (4.6–13.1)
Initiators	NA	2.9 (0.05–5.8)	8.1 (1.6–14.5)
**Recurrent ischemic or hemorrhagic stroke[a]**			
	**Non-fatal (n=85)**		
Non-initiators	8.7 (6.0–11.5)	11.4 (8.2–14.6)	15.2 (11.0–19.3)
Initiators	9.5 (5.4–13.5)	17.1 (11.5–22.8)	18.7 (12.4–25.1)
	**Fatal (n=10)**		
Non-initiators	NA	1.13 (0.004–2.3)	1.13 (0.004–2.3)
Initiators	NA	2.7 (0.3–5.0)	4.2 (0.4–8.1)
	**Non-restroke death (n=70)**		
Non-initiators	NA	3.1 (1.2–5.0)	15.2 (10.1–20.3)
Initiators	NA	4.3 (0.9–7.6)	17.7 (9.3–26.1)

PSAD = post-stroke antidepressant; CI = confidence interval; NA = Not applicable.

[a]Eight PSAD initiators and 16 matched controls were excluded from the vascular event dataset (n=585), and 2 initiators and 4 matched controls were excluded from the recurrent stroke dataset (n=603), since in these sets the PSAD use started after the event of interest.

Supplemental Figure 1. The distribution of propensity score among PSAD initiators (n=206) and non-initiators (n=657) before and non-initiators after (n=403) propensity score matching.

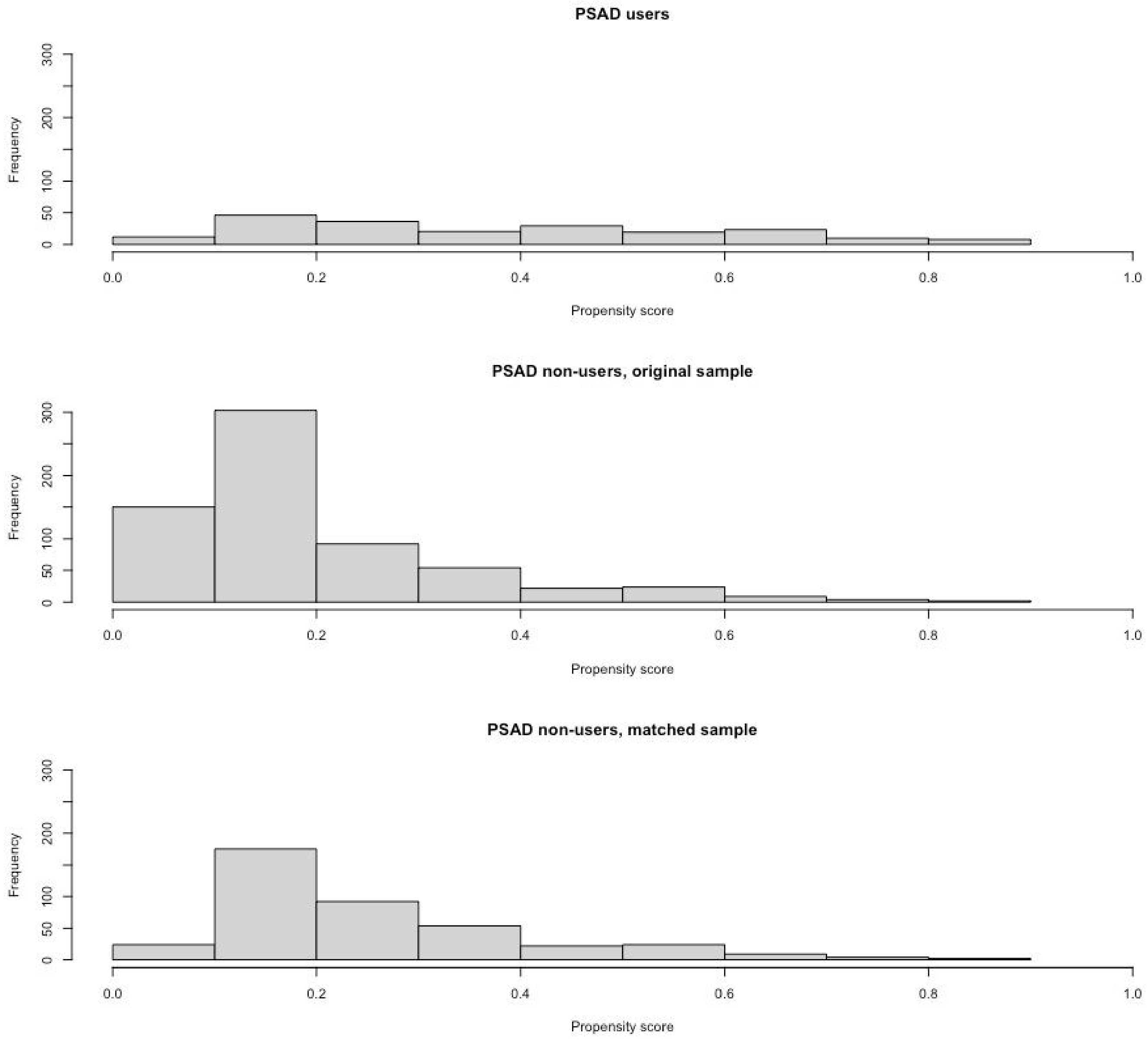

Supplement: Supplemental Material [file IANN_A_2089729_SM4272.pdf]
